# Changes in Physiological and Agronomical Parameters of Barley (*Hordeum vulgare*) Exposed to Cerium and Titanium Dioxide Nanoparticles

**DOI:** 10.3390/ijerph13030332

**Published:** 2016-03-17

**Authors:** Luca Marchiol, Alessandro Mattiello, Filip Pošćić, Guido Fellet, Costanza Zavalloni, Elvio Carlino, Rita Musetti

**Affiliations:** 1 DI4A—Department of Agriculture, Food, Environment and Animal Sciences—University of Udine, via delle Scienze 206, I-33100 Udine, Italy; alessandro.mattiello@uniud.it (A.M.); filip.poscic@uniud.it (F.P.); guido.fellet@uniud.it (G.F.); czavalloni@csustan.edu (C.Z.); rita.musetti@uniud.it (R.M.); 2 Agriculture Studies Department, California State University Stanislaus, One University Circle, Turlock, CA 95382, USA; 3 IOM-CNR Laboratorio TASC, Area Science Park Basovizza, Bld MM, SS 14, Km 163.5, 34149 Trieste, Italy; carlino@iom.cnr.it

**Keywords:** cerium oxide nanoparticles, titanium oxide nanoparticles, barley, plant growth, food chain

## Abstract

The aims of our experiment were to evaluate the uptake and translocation of cerium and titanium oxide nanoparticles and to verify their effects on the growth cycle of barley (*Hordeum vulgare* L.). Barley plants were grown to physiological maturity in soil enriched with either 0, 500 or 1000 mg·kg^−1^ cerium oxide nanoparticles (*n*CeO_2_) or titanium oxide nanoparticles (*n*TiO_2_) and their combination. The growth cycle of *n*CeO_2_ and *n*TiO_2_ treated plants was about 10 days longer than the controls. In *n*CeO_2_ treated plants the number of tillers, leaf area and the number of spikes per plant were reduced respectively by 35.5%, 28.3% and 30% (*p* ≤ 0.05). *n*TiO_2_ stimulated plant growth and compensated for the adverse effects of *n*CeO_2_. Concentrations of Ce and Ti in aboveground plant fractions were minute. The fate of nanomaterials within the plant tissues was different. Crystalline *n*TiO_2_ aggregates were detected within the leaf tissues of barley, whereas *n*CeO_2_ was not present in the form of nanoclusters.

## 1. Introduction

The useful properties of engineered nanoscale materials (ENMs) have resulted in the rapid development of nanotechnologies and large-scale production of nanoparticles or nanoparticles-containing products [1]. The increasing use of ENMs may result in the rise in the flux of ENMs discharged into the environment. Water bodies and soil are assumed to be the primary environmental recipients of nanomaterials [2].

Recent estimates included cerium oxide nanoparticles (*n*CeO_2_) and titanium oxide nanoparticles (*n*TiO_2_) among the 10 most commonly produced ENMs that are used worldwide. In the cosmetic industry, solar cells, paints, cements and coatings about 10,000 t of *n*TiO_2_ are used per year [3]. A number of applications of *n*TiO_2_ are in use in the food industry and agriculture, serving as nano-sensors and nano-agents for new delivery systems of plant protection products and fertilizers [4,5]. Also, *n*CeO_2_ have a broad range of industrial application as additives in glass and ceramics, fuel-cell materials and the automotive industry [6]. On the other hand, *n*CeO_2_ and *n*TiO_2_ are both included in the list of ENMs for immediate priority testing by the Organization for Economic Cooperation and Development [7]. Vascular plants should be of particular concern as they interact closely with the environment and are conduits for bioaccumulation through the food chain [8,9,10,11].

Even though this subject is of primary importance, to date relatively few studies have been carried out on the responses of crops exposed to metal nanoparticles. Most of the currently available papers have reported data collected from experiments performed in hydroponic conditions [6,7,8,9,10,11,12,13]. Such approaches are not able to account for the complexity of the soil-plant system [14].

Early studies concerning the relationships between plants and *n*CeO_2_ were mostly focused on the initial developmental stages of plants. López-Moreno *et al.* [12] observed seed germination and root elongation in cucumber, tomato, alfalfa and corn exposed to 0–4000 mg·L^−1^ *n*CeO_2_ with contradictory results. Ma *et al.* [15] verified that the root growth of cabbage, cucumber, radish, rape, tomato, and wheat were not affected by 2000 mg L^−1^ *n*CeO_2_. At the same concentration, the germination of soybean was undisturbed but indications of genotoxicity were reported by López-Moreno *et al.* [16]. The same authors demonstrated significant species-specific differential levels of plant uptake and translocation of Ce in plants exposed to 4000 mg L^−1^ of *n*CeO_2_. Differences in root microstructures (e.g., pore size in root hairs) and physical and chemical interactions between *n*CeO_2_ and root exudates in the rhizosphere could explain the differences [17]. The movements of *n*CeO_2_ within the root tissues of kidney bean to the aerial tissues were verified by combining ICP-OES and μ-XANES analysis [18]. More recently it was reported *n*CeO_2_ induced compositional modifications in the root xylem in seedlings of rice, wheat and barley [19]. Finally, a life cycle study on barley grown in soil amended with 125–500 μg·g^−1^ *n*CeO_2_, reported both beneficial and harmful effects of nanoceria [20].

The information on the effects of Ti nano-forms are controversial because several papers have demonstrated positive [21,22,23,24,25] or negative [26,27,28] effects of *n*TiO_2_ on plants. Recently, Frazier *et al.* [29] reported that in plantlets exposed to *n*TiO_2_ (range 1000–25,000 ppm) for three weeks the leaf count, root length and plant biomass significantly increased as Ti concentrations were raised. In contrast, Pakrashi *et al.* [30] observed a dose-dependent decrease in the mitotic index and an increase in the number of chromosomal aberrations in root tips of *Allium cepa* exposed to 12.5–100 mg *n*TiO_2_·mL^−1^.

Modelling studies have predicted that ENMs released to the environment are likely to be mostly found in water, sediments, and soils [31,32]. Considering the increasing speed of nanotechnology development it is plausible to assume that different ENMS might be present simultaneously in the environmental compartments (water, sediments, soil and biota). Therefore, living organisms could be exposed to a co-occurrence of EMNs. However, this issue is still poorly explored in literature and we are still lacking systematic and reliable information [33]. Moreover, most studies hitherto have only evaluated crop plants to the germination stages, and have not examined the complete developmental cycle.

Currently, nanotechnology is considered as an important tool in agriculture with the potential to provide new strategies to improve crop production for human consumption and animal feeding and promoting a reduction in the use of pesticides [34]. Since several scientific evidences suggest that nanomaterials may induce harmful environmental effects, it is crucial to investigate on the impact of nanomaterials on crops. In our study barley was considered as model crop since it is among the world’s most important cereal crops [35]. In the present study, barley plants were grown for the whole crop cycle in a soil enriched with different levels of *n*CeO_2_ and *n*TiO_2_ and their combination in a fully factorial design. To the authors’ knowledge, this is the first study that reports data on plants exposed simultaneously to different metal nanoparticles. We hypothesized that (i) the exposure of barley to *n*CeO_2_ and *n*TiO_2_ (also combined each other) would influence plant growth; that (ii) nanoparticles would influence plant physiology and that (iii) the concentration of *n*CeO_2_ and *n*TiO_2_ would affect the uptake of Ce and Ti in roots and the translocation of such elements in the vegetative plant fractions and in seeds.

## 2. Experimental Section

### 2.1. Characterization of nCeO_2_ and nTiO_2_

Cerium (IV) oxide nanopowder and titanium (IV) oxide anatase nanopowder both having a nominal average particle size of 25 nm were purchased from Sigma-Aldrich (St. Louis, MO, USA). Particle characterization was carried out at the Facility for Environmental Nanoscience Analysis and Characterization (FENAC), University of Birmingham (UK).

The specific surface area of the *n*CeO_2_ and *n*TiO_2_ powders was measured by the Brunauer–Emmett–Teller (BET) method by using the Surface Area and Pore Size Analyser SA 3100 plus (Beckman Coulter, Brea, CA, USA). The samples were outgassed at 300 °C for 180 min and the nitrogen adsorption-desorption isotherms were recorded at liquid nitrogen temperature (77K). The BET values were 46.1 m^2^·g^−1^ and 61.6 m^2^·g^−1^ for *n*CeO_2_ and *n*TiO_2_ respectively.

The size distribution of the *n*CeO_2_ and *n*TiO_2_ powders were measured by Atomic Force Microscopy (AFM) method using PSIA XE100 (Park System, Suwon, Korea). The samples were prepared by spreading the *n*CeO_2_ and *n*TiO_2_ powder over a mica sheet pre-treated with poly-L-lysine (Sigma Aldrich). The average height was obtained by measuring at least 100 nanoparticles in non-contact mode. The average height of the *n*CeO_2_ and *n*TiO_2_ powder were 32.6 ± 20.7 nm and 41.8 ± 24.3 nm, respectively. Subsequently, the *n*CeO_2_ and *n*TiO_2_ powders were suspended in deionized water at a concentration of 1000 ppm, sonicated, and simultaneously heated at 60 °C for 30 min. The suspensions were characterized for z-average size, measured as hydrodynamic diameter, and zeta potential, via electrophoretic mobility, by dynamic light scattering (DLS) method using the Nano ZS90 (Malvern Instruments, Malvern, UK). The z-average size of *n*CeO_2_ and *n*TiO_2_ powder was 174 ± 1.19 and 925 ± 105 nm, respectively. The zeta potential of the *n*CeO_2_ and *n*TiO_2_ powders were respectively 0.027 ± 0.064 mV and 19.9 ± 0.55 mV. Finally, the *n*CeO_2_ and *n*TiO_2_ powders suspension were characterized also with the Differential Centrifugal Sedimentation (DCS) method using CPS DC24000 UHR (Analytik, Swavesey, UK). The average sizes were 188 ± 5.9 nm and 690 ± 17.5 nm, respectively for *n*CeO_2_ and *n*TiO_2_.

### 2.2. Addition of Nanoparticles to Soil

The soil used for this study was collected in Udine, Italy (46°04′52′′ N, 13°12′33′′ E; top 20 cm) air dried at room temperature and sieved through a 2 mm mesh prior to characterization. The soil was classified as clay soil (sand 26%, silt 6.4% and clay 67.6%) with pH 7.4, cation exchange capacity (CEC) of 13.9 (cmol·kg^−1^ DM), electrical conductivity (EC) of 1235 (μS·m^−1^) and organic matter (OM) content of 4.4%.

Eight mixtures of soil and *n*CeO_2_ and *n*TiO_2_ were prepared following the procedure used by Priester *et al.* [36]. The main treatments were made by adding *n*CeO_2_ and *n*TiO_2_ powders directly to the soil and mixing it in a portable concrete mixer previously sealed, to obtain a concentration of 2000 mg·kg^−1^ of either *n*CeO_2_ and *n*TiO_2_. The *n*CeO_2_/*n*TiO_2_ amended soils were stored in the dark at 10 °C for two weeks. After soil equilibration the final doses of 500 and 1000 mg·kg^−1^, respectively (Ce 500, Ce 1000, Ti 500, Ti 1000) were prepared by serial dilution with soil. Four additional treatments were obtained by combining the stock soils to achieve the following combinations: *n*CeO_2_ 500 mg·kg^−1^/*n*TiO_2_ 500 mg·kg^−1^ (Ce 500-Ti 500), *n*CeO_2_ 500 mg·kg^−1^/*n*TiO_2_ 1000 mg·kg^−1^ (Ce 500-Ti 1000), *n*CeO_2_ 1000 mg·kg^−1^/*n*TiO_2_ 500 mg·kg^−1^ (Ce 1000-Ti 500) and *n*CeO_2_ 1000 mg·kg^−1^/*n*TiO_2_ 1000 mg·kg^−1^ (Ce 1000-Ti 1000). After another soil equilibration round of three days, five microcosms (4 L polyethylene pots) per treatment were filled with the control and nanoparticles amended soil (*n* = 45). The control treatment received no nanoparticle amendment.

### 2.3. Plant Growth and Harvest

Eight seeds of spring barley (*Hordeum vulgare* L., *cv*. Tunika) obtained from S.I.S Società Italiana Sementi (San Lazzaro di Savena, Bologna, Italy) were sown in microcosms containing the *n*CeO_2_/*n*TiO_2_ amended soils. The trial was carried out in a semi-sealed greenhouse under full sunlight. Two weeks after seed planting, the seedlings were thinned to four seedlings per microcosm. At tillering, two plants per pot were removed, therefore 90 plants were observed during the experiment (ten plants per treatment). During the growth period the microcosms were irrigated to maintain the soil at 60% of water holding capacity (WHC). During the barley growth cycle the microcosms were singularly weighed and irrigated to compensate for evapotranspiration. Phenological stages were monitored by adapting the Decimal Growth Scale [37] throughout the growth cycle and were based on 50% of plants within the treatments at each stage. Plants were harvested at physiological maturity. Prior to collecting plants, plant height was measured from soil surface to the flag leaf using a standard meter stick (1 m). Then plant shoots were severed at the collar with a razor blade and then separated into stems, leaves, spikes, and grains. Leaf area was measured using a LI-3100C Area Meter (Li-Cor Corporation, Lincoln, NE, USA). Plant samples were thoroughly washed in tap water and rinsed three times with distilled water. In addition, roots were washed in 400 mL of 0.01 M of nitric acid in a shaker bath at 300 rpm for 5 min to remove metal ions adsorbed at the surface. The plant fractions were oven dried at 105 °C for 24 h and weighed.

### 2.4. Gas Exchange Parameters

The photosynthetic rate at saturating light intensity (A_max_, μmol CO_2_·m^−2^·s^−1^), transpiration rate (T_r_, mmol H_2_O·m^−2^·s^−1^), and stomatal conductance (g_s_, mol air m^−2^·s^−1^) were measured with a portable gas exchange system (LI-6400, LI-COR, Inc. Lincoln, NE, USA). The gas exchanges measurements were carried out on the flag leaf at booting, heading and milk maturity on three individual plants per treatment. The measurements were made after allowing leaves to reach steady-state conditions at saturating photosynthetic active radiation (PAR, 1200 μmol·m^−2^·s^−1^), at a CO_2_ concentration of 400 ppm, and at a temperature of 25 °C, and were collected between 9 and 14 h with evaluation of five measurement periods at intervals of 7–8 days.

### 2.5. TEM Observations

A small leaf portion (2 × 3 mm) was excised close to the central vein of the youngest leaf before the emergence of flag leaves. The fresh samples were fixed for 2 h at 4 °C in 0.1% (wt/vol) buffered sodium phosphate and 3% (wt/vol) glutaraldehyde at pH 7.2. They were then post-fixed with 1% osmium tetroxide (wt/vol) in the same buffer for 2 h, dehydrated in an ethanol series, and embedded in Epon/Araldite epoxy resin (Electron Microscopy Sciences, Fort Washington, PA, USA). Serial ultrathin sections from each sample were cut with a diamond knife, mounted 100/200 folding grids, and then observed under a Philips CM 10 transmission electron microscope (TEM, FEI, Eindhoven, The Netherlands) operating at 80 kV.

### 2.6. Spectroscopy Analysis

Samples of soils were oven-dried (105 °C for 48 h) and digested in 11 mL of a 10 to 1 (*v*/*v*) mixture of 96% (*v*/*v*) sulphuric acid and 30% (*v*/*v*) hydrogen peroxide in Teflon cylinders for 20 min at 200 °C in a microwave oven (MARS Xpress, CEM, Matthews, NC, USA). After digestion, samples were diluted 1 to 20 with milliQ water, filtered through 0.45 µm filters and Ce and Ti were determined with an ICP-OES (Vista MPX, Varian Inc., Palo Alto, CA, USA). Oven-dried plant fractions were digested in 10 mL of a 1 to 4 (*v*/*v*) mixture of 65% (*v*/*v*) nitric acid and 30% (*v*/*v*) hydrogen peroxide in Teflon cylinders for 10 min at 175 °C in microwave oven (CEM MARS Xpress). The plant extracts were filtered with Whatman^®^ PTFE membrane filters (0.45 µm), diluted, and analysed. Total Ce and Ti in roots, stems, and leaves were determined by an ICP-OES using yttrium as internal standard. The Ce and Ti contents in kernels were quantified using an ICP-MS (Aurora M90, Bruker, Bremen, Germany) with an internal standard solution of ^72^Ge and ^89^Y. Quality control for both ICP-OES and ICP-MS was carried out using reagent blank samples, and triplicates reading for each sample. Certified standard reference material (NIST 1573a Tomato leaves) was analysed to validate the protocol.

### 2.7. TEM X-ray Microanalysis

Crystal structure and chemistry of the nanoparticles were studied by using a 2010F UHR TEM/STEM (JEOL, Peabody, MA, USA) equipped with a low spherical aberration coefficient (Cs = 0.47 ± 0.01 mm) objective pole piece and energy dispersive X-ray spectrometer (EDXS). The experiments were performed at an accelerating voltage of 200 kV corresponding to an electron wavelength of 2.5 pm. The EDXS spectra were acquired in scanning transmission electron microscopy (STEM) configuration by rasterizing an electron probe of 0.5 nm within the area of interest imaged by a high angle annular dark field (HAADF) detector to accurately determine the chemical assessment of the investigated nanoparticles. Nanodiffraction were acquired in TEM by illuminating the area of interest with a 50 nm parallel electron probe to study the crystal features of individual nanoparticles [38].

### 2.8. Data Analysis

The microcosm study was arranged in a completely randomized factorial design with nine treatments (control soil, two levels of *n*CeO_2_, two levels of *n*TiO_2_ and four *n*CeO_2_/*n*TiO_2_ mixtures) and five replicates. Data were tested for homoscedasticity and normality using the Bartlett’s test and the Shapiro–Wilk test, respectively. The differences were statistically significant, as determined by one-way and two-way analysis of variance (ANOVA). Tukey’s Multiple Comparison test (*p* = 0.05) in case of significant effects were used to analyse individual effects. Statistical analysis was performed using the SPSS program (ver. 16, SPSS Inc., Chicago, IL, USA).

## 3. Results

### 3.1. Phenology and Growth of Barley

**Figure 1:**
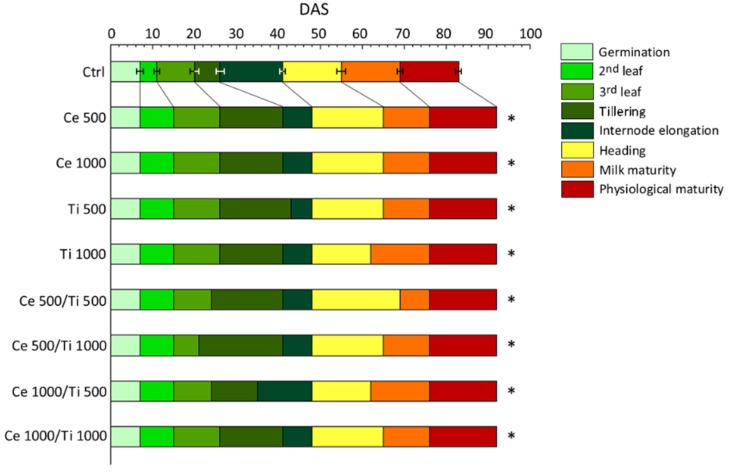
Cumulative contribution of vegetative and reproductive phenophases to the phenology of plants of barley grown in control soil and *n*CeO_2_ and *n*TiO_2_-amended soil. DAS = days after sowing. Error bars represent ± standard error. * denote significant differences with respect to control (*p* ≤ 0.05).

A week after sowing, all the plants had germinated, apparently unaffected by the presence of metal oxide nanoparticles in the soil and without early symptoms of phytotoxicity in treated plants. From the 2nd leaf stage we observed that *n*CeO_2_ and *n*TiO_2_ treated plants had a longer vegetative period than the controls (Figure 1).

This delay was also verified at the 3rd leaf and tillering stages, even if it was less pronounced for the Ce 500/Ti 1000 treatment. The delay in the nanoparticle-treated plants reached its maximum extent (about ten days) at heading and during the ripening stages (Figure 1). Milk maturity was reached on average 65 days after sowing (DAS) in treated plants. Ti 1000 and Ce 1000/Ti 500 plants were the earliest (62 DAS) and the Ce 500/Ti 500 ones were the latest (69 DAS) at entering physiological maturity (Figure 1).

**Figure 2:**
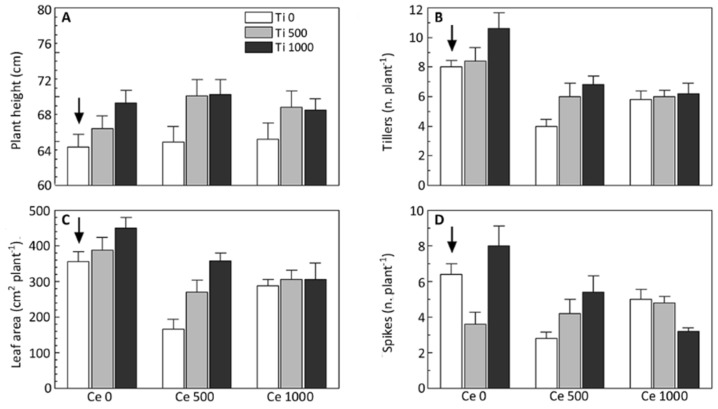
Plant height (**A**); number of tillers (**B**); leaf area (**C**) and number of spikes (**D**) observed in plants of barley grown in control soil and *n*CeO_2_ and *n*TiO_2_-amended soil. Bars are mean standard error (*n* = 5). Arrows indicate the control.

A two-factor analysis of variance (ANOVA) was performed on the following variables: plant height, number of tillers, leaf area per plant and number of spikes. Among the morphological traits that we considered, plant height was the least sensitive to the *n*CeO_2_ and *n*TiO_2_ treatments. The interaction *n*CeO_2_ × *n*TiO_2_ was not significant (Figure 2A). The plants treated with *n*TiO_2_ were significantly taller than the others were (*p* = 0.0035), whereas there was no statistically significant effect of *n*CeO_2_.

The formation of secondary shoots was significantly influenced, but in an opposite way, by the experimental factors. The interaction effect between *n*CeO_2_ and *n*TiO_2_ on tiller’s formation was not statistically significant. The number of tillers per plant was significantly stimulated by *n*TiO_2_, compared to the control (*p* = 0.009). In fact, in Ce 0 plants *n*TiO_2_ 1000 promoted, on average, the formation of 2.60 secondary shoots more (+25%) than the control plants (*p* = 0.032); a more pronounced effect (2.80 tillers more than controls) was recorded in Ce 500 plants (*p* = 0.026) (Figure 2B). In contrast, *n*CeO_2_ had a statistically significant (*p* < 0.001) negative effect on tiller formation (Figure 2B). In particular, on average in Ce 500 and Ce 1000 plants the number of secondary shoots was about 35% lower than both control plants (*p* = 0.002) and Ti treated ones (respectively *p* = 0.034, and *p* < 0.001).

The number of tillers and the plants’ total leaf area are closely linked. In fact, the ANOVA showed a significant effect of both *n*CeO_2_ (*p* < 0.001) and *n*TiO_2_ (*p* = 0.001). The interaction *n*CeO_2_ × *n*TiO_2_ was not significant. Multiple comparisons were run for each simple main effect. As noted earlier, in absence of Ti, Ce 500 had a strong negative effect on plant vegetative growth, in fact the average leaf area per plant was about one-half that of the control (166 *vs.* 356 cm^2^ of leaf surface per plant) (*p* < 0.001) (Figure 2C). Such a negative influence of *n*CeO_2_ in Ce 500 plants appeared to have been mitigated by *n*TiO_2_ which had a positive significant effect (*p* < 0.001) (Figure 2C). In regards to the plant’s response to *n*CeO_2_/*n*TiO_2_ mixtures, although the interaction was not statistically significant (*p* = 0.091), we assumed that *n*TiO_2_ at respectively 500 and 1000 mg per kg of soil was able to remediate the adverse impact on leaf growth of *n*CeO_2_ (Figure 2C).

One of the main yield components in barley is the number of spikes per plant. Two-way ANOVA revealed a statistically significant effect of the main factor *n*CeO_2_ (*p* = 0.0116) and a significant interaction between *n*CeO_2_ and *n*TiO_2_ (*p* = 0.0016). The effect *n*TiO_2_ in this case was not statistically supported, being likely hidden by data variability (Figure 2D). The mean number of spikes in Ce 500 and Ce 1000 plants was 4.2 corresponding to a reduction of 38% than the controls (2D). Regarding interaction, multiple comparisons of the means showed the different main effects of *n*CeO_2_ and *n*TiO_2_. In absence of *n*TiO_2_ (Ti 0), the number of spikes per plant were negatively affected by *n*CeO_2_. There was a statistically significant difference (*p* = 0.003) between Ce 0 and Ce 500 plants (6.4 and 2.8 spikes per plant, respectively; −56%). The negative influence of *n*CeO_2_ was confirmed at the highest dose (Ce 1000) even though it was lighter in magnitude (22% lower than control plants) and not statistically different from Ce 500 (Figure 2D). The same effects were verified also in Ti 1000 plants, where both the levels of *n*CeO_2_ determined a reduction of spike number of 32.5% and 60% respectively for Ce 500 (*p* = 0.033) and Ce 1000 (*p* < 0.001). The intermediate dose of *n*TiO_2_ (500 mg·kg^−1^) negatively affected the number of spikes in plants (−43.7% than controls, *p* = 0.020). On the contrary, Ti 1000 plants had 8 spikes each (higher that control plants although not statistically significant). In other words, in terms of spike formation our data suggest that, in absence of *n*CeO_2_, the higher dose of *n*TiO_2_ was, at least, harmless to plants. This evidence is in contrast with other growth parameters. Moreover, we detected a negative *n*TiO_2_ dose-effect on plant tillers’ number and leaf area (Figure 2B–D).

### 3.2. Gas Exchanges

The leaf photosynthetic rate (A_max_), stomatal conductance (g_s_) and transpiration rate (T_r_) at three different growth stages (booting, heading, milk maturity) are shown in Table 1. Because the treatments affected plant development by causing a shift in phenological stages, the gas exchange parameters were evaluated by comparing plants using the same phenological phase.

Both *n*CeO_2_ and *n*TiO_2_ treatments had a statistically significant effect on the photosynthetic parameters, whereas their interaction was not significant at any of the growth stages. Table 1 reports the data regarding the main factors.

At the booting phase, A_max_ and T_r_ were positively affected by Ce 500 compared to control plants (respectively +26% and +75%). However, Ce 1000 plants behaved like the controls suggesting that the maximum concentration for a beneficial effect from *n*CeO_2_ had been exceeded (Table 1). As expected, in the subsequent phases of the life cycle of barley A_max_ declined, with no evidence of statistically significant differences between treatments and control. At booting Ti 1000 had an overall positive effect compared to the control plants: A_max_, gs, and T_r_ significantly increased by 37, 89, and 92%, respectively. The Ti 500 treatment had an intermediate effect (Table 1).

### 3.3. Plant Uptake and Accumulation of Cerium and Titanium

Table 2 and Table 3 present the concentrations of Ce and Ti in the roots, stems, leaves, and kernels of barley. In general, the concentration of Ce and Ti in the plant tissues showed a dose-response.

A statistically significant dose-response in Ce concentration was recorded in all plant fractions with the exception of kernels. In Ce 500 and Ce 1000 plants, the mean levels of Ce in the roots were 45.3 mg·kg^−1^ and 96.9 mg·kg^−1^, respectively (Table 2). The significant dose-dependent response in root Ce accumulation was confirmed also in *n*CeO_2_/*n*TiO_2_ treatments.

Ce concentration in stems was significantly different between Ce 500 and Ce 1000 plants both in the case where Ce was individually supplied or when it was associated with the Ti. The Ce root to shoot translocation percentage in treated plants ranged between 1.24 and 9.1, respectively, for Ce 1000 and Ti 500, indicating that Ce accumulation in the aboveground plant fractions occurred at very low magnitude. The highest Ce concentration in leaves was recorded in Ce 1000 plants (3.03 mg·kg^−1^). As expected, in the leaves of Ctrl plants and Ti 500 and Ti 1000 ones, lower Ce accumulation values (0.73, 0.77 and 0.84 mg·kg^−1^, respectively) were observed.

Despite the increase in concentration of Ti in the soil (*p* = 0.0001), due to the addition of *n*TiO_2_, the Ti uptake and accumulation in the plant fractions did not respond to the treatment. No statistically significant differences in Ti concentration in roots, as well as in stems and leaves, were observed (Table 3). As for Ce, in the case of Ti the root-to-shoot translocation percentage was very low, ranging between 1.42 and 1.91.

Barley grains are used as food for humans and animals, as well as for other applications (e.g., malting and flour). Thus it is appropriate to examine whether nanoparticles are able to reach the kernels during ripening (Table 2 and Table 3). No statistically significant differences among the treatments were recorded. In absolute values, the content of both elements in the kernels was three-four orders of magnitude lower than those recorded in plant leaves (Ce 0.718 μg·kg^−1^ and Ti 1.77 μg·kg^−1^, respectively in Table 2 and Table 3).

### 3.4. Ultrastructural Analyses

**Figure 3:**
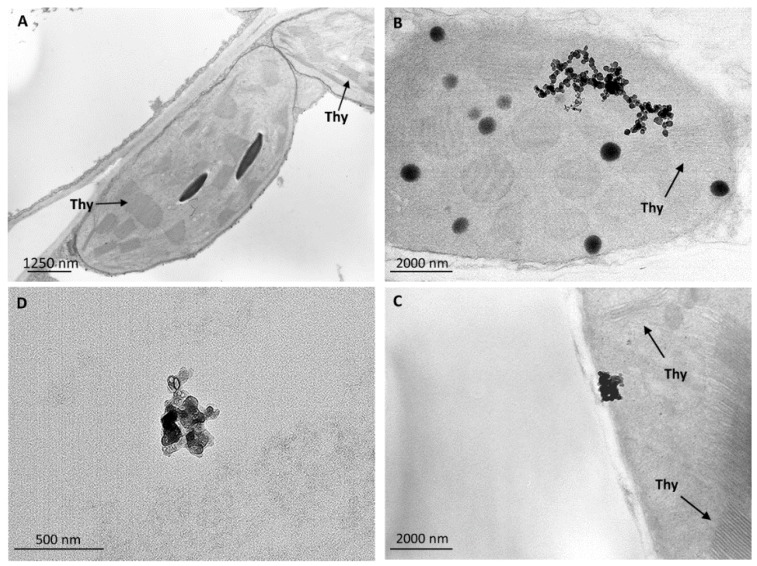
Representative TEM micrographs of leaf tissues from barley plants. In control untreated leaf tissues (**A**) nanoparticles are absent and chloroplast ultrastructure is well preserved. In plants grown in *n*CeO_2_ and *n*TiO_2_-amended soil clusters of nanoparticles are visible in the stroma of the chloroplasts (**B**, Ce 1000; **C**, Ti 1000) and in the vacuoles of parenchymal cells (**D**, Ce 1000). Chloroplast structure seems not affected by nanoparticle treatment (arrows in **A**, **B** and **C** indicate thylakoids).

**Figure 4:**
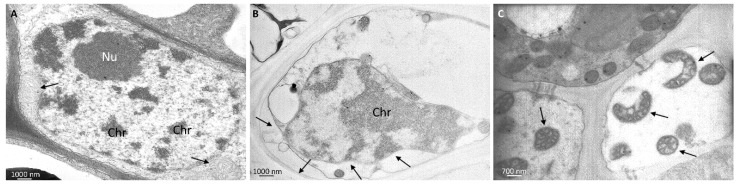
Representative TEM micrographs of leaf tissues from barley plants. (**A**) In control untreated vascular parenchyma cells mitochondria (arrows) and nuclei appear well preserved with regular shape and intact membranes. Nucleolus (Nu) is recognizable and chromatin (Chr) is normally dispersed. In plants grown in *n*CeO_2_ and *n*TiO_2_-amended soil vascular parenchyma cell has detached plasma membrane (arrows) and nucleus presents lobed shape and condensed chromatin (Chr) (**B**, Ce 1000). Mitochondria have disorganized, swollen christae (arrows in **C**, Ce 1000).

**Figure 5:**
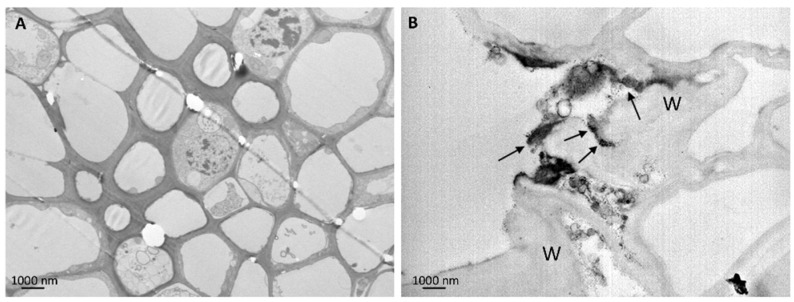
Representative TEM micrographs from secondary vein tissues of barley plants. (**A**) Control untreated vein tissues appear well structured, cell walls are regular in shape and thickness; (**B**) Ti 1000: secondary veins showed cells with contorted cell walls (W) associated with little dark precipitates (arrows).

To verify the root uptake and subsequent translocations of *n*CeO_2_ and *n*TiO_2_ from roots to aerial plant fractions, ultrastructural analyses on plant leaf tissues were carried out. Nanoparticles were not present in untreated control leaf tissues, which presented well preserved ultrastructure and organelles (Figure 3A; Figure 4A; Figure 5A). Rare clusters of nanoparticles were found in leaves sampled from plants grown in soil enriched with the different combinations of *n*CeO_2_ and *n*TiO_2_, at both concentrations (Figure 3).

Nanoparticles were observed in parenchyma leaf tissues, in the stroma of the chloroplast and in the vacuoles, (Figure 3B–D). Despite the presence of *n*CeO_2_ and *n*TiO_2_, the chloroplasts appeared normal, with preserved ultrastructure, and, in general, the cell compartments of the chlorophyll parenchyma did not appear affected by the treatments (Figure 3B). This evidence was in agreement with phenotypical/morphological analyses, as we did not observe macroscopic cell death at the tissue level after the *n*CeO_2_ and *n*TiO_2_ treatments. Nevertheless, at the vascular tissue, some ultrastructural modifications were visible, especially those affecting the cellular organelles: some nuclei showed condensed chromatin, mitochondria swollen cristae (Figure 4B,C). Only in Ce 1000 and Ti 1000 leaf tissues, few secondary veins showed irregular-shaped cells with contorted walls (Figure 5B).

### 3.5. Nanostructures in Leaf Tissues

**Figure 6:**
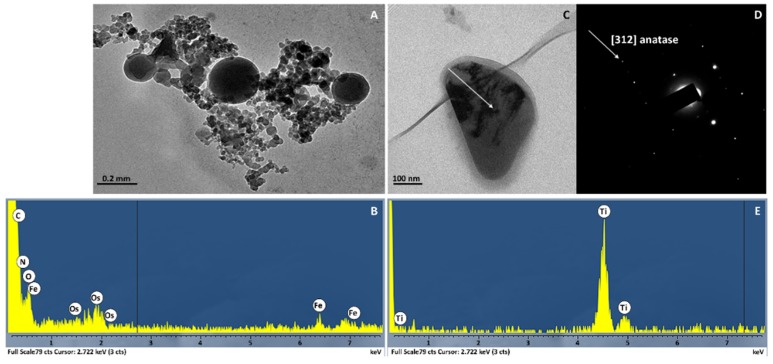
(**A**) Brightfield TEM image of representative nanostructures observed in Ce 1000 leaves; (**B**) EDXS spectrum as acquired in the particle area shown in brightfield mode; (**C**) Brightfield TEM image of Ti nanostructures in Ti 1000 leaves; (**D**) Nanodiffraction pattern as acquired on individual nanostructures indicated by the arrow in the brightfield image (see text); (**E**) EDXS spectrum as acquired on the region indicated in the brightfield image.

After verifying the root-to-leaves translocation of Ce and Ti, STEM EDXS observations were carried out to detect the presence of *n*CeO_2_ and *n*TiO_2_ within the leaf tissues. Regarding *n*CeO_2_, several nanostructure aggregates were observed, with sizes ranging from a few nanometers to some hundreds of nanometers. Interestingly, nanodiffraction measurements revealed an amorphous structure in most of cases. The compositional analysis by EDXS reported in Figure 6 did not show Ce in such aggregates so Ce was unlikely to be present in the form of nanoclusters within the leaf tissues of barley.

In Figure 6A, a brightfield image shows a typical aggregate of clusters with large size dispersion. No Bragg’s peaks were observed in the relevant nanodiffraction and the EDXS spectrum showed no evidence of Ce characteristic X-rays, but only those of light elements (Figure 6B). Even though we lack direct experimental evidence, it is very likely that the small amount of Ce in leaves (Table 3) is aggregated in ionic form to organic molecules. It is also worthwhile mentioning that during extensive TEM/STEM sessions, isolated crystalline *n*CeO_2_ were observed in only two cases. Therefore, we conclude that *n*CeO_2_ induces a massive formation of amorphous clusters of light elements rather than nanometer-scale clusters of CeO_2_. Such aggregates were abundant in the leaves of Ce 1000 plants but absent in control ones (Figure 5).

Several aggregates of nanoparticles were observed in the leaves of plants exposed to *n*TiO_2_. In Figure 6C–E, a representative result of the chemical and structural analysis performed on a Ti aggregate is shown. Figure 6D shows a brightfield image of several dark aggregates. The nanodiffraction pattern acquired demonstrates the crystalline nature of the aggregates (Figure 6D). The measured lattice spacing is compatible with TiO_2_ crystals. Indeed, the diffraction intensities belong to TiO_2_ nanocrystallites with different orientation with respect to the primary electron beam. As an example, the arrow in the pattern points to the systematic reflection of a TiO_2_ anatase particle oriented close to the [312] zone axis (Figure 6D). To identify the chemical signature of the element contained in the nanostructures, the EDXS spectrum was acquired in the same area of the nanodiffraction, and the emission of characteristic fluorescence X-rays of Ti atoms was recorded (Figure 6E). In conclusion, we report the presence of TiO_2_ nanoclusters in parenchyma leaf tissues—particularly inside the chloroplasts—of barley plants exposed to *n*TiO_2_.

## 4. Discussion

Model simulations have demonstrated that flows of ENMs are currently able to reach natural ecosystems [39]. For this reason, questions are rising about the consequences of the interaction of ENMs with biota. Plant phenological traits and growth parameters always respond to the physical environment, so they can be used to assess the adaptive behaviours of the plants and evaluate their relationships to the ecosystem where they grow.

It was reported that ENMs have the potential to influence the growth of some crops with enhancing or inhibitory effects on plant growth according to their composition, size and physical and chemical properties [10,36,40].

Studying the phenological stages of barley plants, according to the findings of Rico *et al.* [20] and Yoon *et al.* [40], we verified that *n*CeO_2_ and *n*TiO_2_ treated plants had a longer vegetative period than the controls. This fact *per se* may not be negative. In fact, a longer vegetative phase may promote higher biomass and grain yield because plants are allowed to produce more photosynthetically active leaves and therefore more photosynthates [41].

Our data showed that *n*TiO_2_ were associated with effects opposite to those induced by *n*CeO_2_. First, we observed an *n*TiO_2_ dose-response effect on vegetative growth. Second, the compensation of the adverse effects of *n*CeO_2_ observed in plants grown in *n*CeO_2_/*n*TiO_2_-treated soils was probably due to the beneficial effects of *n*TiO_2_ on plant metabolism. Several evidences obtained in different experimental conditions support this hypothesis. Studies carried out on *Spinacia olearacea* have demonstrated that *n*TiO_2_ promote plant photosynthesis increasing light absorbance and transformation of light energy [42] and enhancing Rubisco activity [22]. Also, it was demonstrated that *n*TiO_2_, could significantly improve CO_2_ fixation by plants, where it enhances absorption and transmission of the solar energy into the chain electron transport in chloroplasts [43]. Our data support such evidences, even though we cannot provide a physiological explanation. On the other hand, working with *Cucurbita pepo* grown in soil containing 400–800 mg·kg^−1^ of *n*CeO_2_, Zhao *et al.* [44] did not observe any differences in the photosynthetic rate between treated plants and controls. Such different responses suggest that, at least at the intermediate level of concentration, different interactions with metabolism occur in various species exposed to metal nanoparticles. However, we still lack a systematic study on the effects on basic metabolism of plants induced by different nanomaterials and different levels of exposition.

At the end of the plant life cycle we studied a number of biometric variables observing some differences between treated plants and the controls. Recently, observations made on plants of barley grown for the entire life-cycle in a soil amended with 125–250–500 mg·kg^−1^ *n*CeO_2_ were reported [20]. Thus, we can compare part of our data with those presented in that paper. In general, our results regarding the effects induced by *n*CeO_2_ on plant growth are in contrast with Rico *et al.* [20]. One of the most relevant differences was that in our case all the plants were able to reach the reproductive stage, whereas Rico *et al.* [19] did not observe the formation of spikes in *n*CeO_2_ 500 mg·kg^−1^ treated plants. Moreover, they observed that *n*CeO_2_ 500 mg·kg^−1^ increased the height of barley plants and the accumulation of dry matter in the shoots, whereas in our case the vegetative growth was not stimulated by any level of *n*CeO_2_. If we consider studies carried out on other crop species, our data partially agree with the findings of Priester *et al.* [36] in *Glycine max*, whereas Zhao *et al.* [44] did not observe statistically significant differences in biometric parameters in plants of *Cucumis sativum* between control plants and those treated with *n*CeO_2_.

With regard to Ce and Ti accumulation in the plant tissues, it must be emphasized that both DLS and DCS analyses, carried out for the higher *n*CeO_2_ and *n*TiO_2_ test suspension (1000 ppm), indicate agglomeration of nanoparticles. Therefore, the actual bioavailability of nanomaterials may be lower than expected. For this reason, we believe that the concentrations of Ce and Ti in the plant tissues were underestimated. In addition, similarly to what happens in the case of conventional contaminants, soil pH, OM, texture and CEC have an important influence on the fate of nanomaterials in soil, [45,46] and particularly with respect to those, which release ions, such as *n*CeO_2_ [47].

It was demonstrated that after root uptake, Ce was able to reach the plant leaves by moving through the vascular system [44]. We verified that Ce did not translocate easily since only a small fraction of the element moved from the roots to the aerial biomass of plants. Our findings and data agree with those obtained in similar studies respectively on soybean, cucumber, and wheat [37,44,48].

The increase in Ti concentration in the roots of Ce 500 and Ce 1000 plants could be due to a stimulatory effect of Ce on root growth and the formation of adventitious roots [49]. Therefore, *n*CeO_2_ treated plants were able to explore a greater volume of soil, accumulating more Ti and Ce compared to the controls. This raises interesting questions about the bioavailability of Ti, which occurs in the soil as *n*TiO_2_ from anthropogenic sources. It was previously observed that an increase in root assimilation and translocation of Ti after exposure to *n*TiO_2_ would mean that the nano formulation of Ti makes it more bioavailable [50]. Other studies have shown that plants could translocate *n*TiO_2_ into their aerial fractions. Titanium nanoparticles of 5 nm in diameter were found in leaves of *Arabidopsis thaliana* [51], in addition both the uptake and translocation of 100 nm *n*TiO_2_ to the leaves of *Nicotiana tabacum* were observed [52]. However, the soil environment is very different and much more complex than artificial conditions. In our case, we observed very different results.

Macro- and micro-morphological observations indicate that under our conditions *n*CeO_2_ and *n*TiO_2_ induced limited cell injuries, at least in the parenchyma tissues. Inside the tissues, metal nanoparticles, depending on their type, shape and concentration may cause either cell death or other side effects [53]. Alternatively, metal nanoparticles can be well tolerated by the cells; however, this does not mean that nanoparticles do not affect cellular pathways [54]. The primary ultrastructural alteration we detected in leaf tissues of plants treated with metal oxide nanoparticles was the condensed chromatin in the nuclei and swollen mitochondria of vascular parenchyma cells. Condensed chromatin and fragmented nuclei, as well as swollen mitochondria, are described in programmed cell death (PCD), reported in cell response to different environmental stimuli and stresses, induced by pathogens [55] and abiotic factors as salinity, cold stress, waterlogging, or hypoxia [56,57].

Referring to the literature findings [26,58,59] we would have expected to find crystalline forms of both elements in the plant tissues. Several nanostructures were observed in the leaf tissues of *n*CeO_2_ treated plants; however, microanalysis did not confirm the presence of Ce in such aggregates. This is in contrast with Zhao *et al.* [58] which verified the presence of *n*CeO_2_ aggregates within vascular tissues of corn, thus demonstrating that Ce nanoparticles migrate through the xylem under transpiration. In studies on soybean plants grown in *n*CeO_2_ amended soil, it was observed that most of the Ce stored in the pods was in the form of *n*CeO_2_ [59].

We have not investigated the speciation of Ce and Ti in plant tissues, however this evidence constitutes a rather strong indication of Ce biotransformation. The biotransformation of ceria nanoparticles in plant tissues was demonstrated in soybean [16] and in cucumber [60,61]. In our case we hypothesized that the formation of the amorphous clusters could be related to the presence of intracellular Ce and, a defensive mechanism against Ce-cytotoxicity, as previously demonstrated [62]. To the contrary, Ti nanoparticles were able to cross biological barriers in plant tissues. In fact, we report the presence of Ti nanoclusters in parenchymatic cells of barley leaves. Early evidence of root to shoot translocation of *n*TiO_2_ was found in hydroponically grown cucumber seedlings, using micro X-ray fluorescence (μ-XRF) and micro X-ray absorption spectroscopy (μ-XANES) [63]. Subsequently, the same evidence was confirmed in older cucumber plants growing in *n*TiO_2_-enriched soil, as well [64]. Our work undertaken with the same method agrees with these findings. Furthermore, unlike what was observed for Ce, we did not observe evidence of biotransformation, confirming the literature findings [64].

## 5. Conclusions

ENMs are currently considered as an emerging class of environmental contaminants and thus, as mentioned above, neither (i) potential interactions with other pollutants [65,66] and/or (ii) the possibility of co-occurrence of ENMs in the environment can be excluded [67]. In other words, there exists a chance of simultaneously exposure of target organisms to different types of ENMs. This implies that the horizon of knowledge gaps on the relationships between ENM and biota has to be moved farther. According to Kumar *et al.* [67] “to properly incorporate exposure of more than one type of ENM, data on toxicity due to mixture of ENMs for a given target organ are required”.

With regard to plants, it is very likely that several research groups are currently working on this topic. However, in literature we found only one paper reporting evidence of stimulating effects on germination and early growth of *Glycine max* induced by a mixture of *n*SiO_2_ and *n*TiO_2_ [68]. Therefore, we reiterate that this paper is the first to report data on the effects of a co-exposure to different metal oxide nanoparticles on a worldwide important crop. Moreover, our data were collected at the end of the life-cycle of soil grown barley plants.

Data available on the effects of ENMs in humans, crop plants, and livestock are not enough to allow for a thorough evaluation of their potential and of their safety. With respect to the aims of this study, although no visual symptoms of toxicity have been detected in plants, we demonstrated that the phenology and growth of barley were affected by *n*CeO_2_ and *n*TiO_2_. All plants concluded their life cycle producing seeds. However, in treated plants we verified differences in some biometric parameters compared to the control ones. In particular, *n*CeO_2_ at the lower concentration were associated with a reduction in the leaf area, the number of tillers and spikes per plant, and for this reason the number of kernels per plant. It can be assumed that this will lead to a reduction in crop production. An attenuation of such adverse effects was observed in plants treated with the higher dose of *n*CeO_2_. Titanium nanoparticles were associated with positive effects on plants. First, we observed *n*TiO_2_ positive dose-response effect on vegetative growth. Second, in plants co-exposed to *n*CeO_2_ and *n*TiO_2_, it is likely that the beneficial effects of Ti on plant metabolism have more than compensated for the adverse effects of Ce. Lacking literature data, at this moment we cannot discuss further these results and this part of our experiments should be considered simply exploratory. However, we demonstrated that the co-occurrence of *n*CeO_2_ or *n*TiO_2_ in soil determined in barley plants effects other than those observed in plants exposed separately to nanomaterials. It is likely that the study on this issue will be further dealt in the near future, through developing appropriate experimental protocols to study the physiological bases of plant response to ENMS co-occurrence.

From an ecological point of view, our data suggest that the fate of *n*CeO_2_ and *n*TiO_2_ could be different. In both cases, their bioaccumulation in plants is minute. Cerium nanoparticles inside plant tissues seem to dissolve into the ionic form that most likely undergoes a subsequent biotransformation. Titanium oxide nanoparticles are found in crystalline form in the leaves of barley and also in the seeds, although in small concentrations, so in this form the *n*TiO_2_ may be able to continue on through the food chain. Further research should be carried out on the intricate relationships that exist in the soil-plant system with respect the fate of nanomaterials.

## Figures and Tables

**Table 1:** ANOVA *p* value for the main factors (*n*CeO_2_, *n*TiO_2_) and interaction (*n*CeO_2_ × *n*TiO_2_) for leaf photosynthetic rate at saturating light intensity (Amax, µmol CO_2_·m^−2^·s^−1^), stomatal conductance (g_s_, mol air·m^−2^·s^−1^) and transpiration rate (Tr, mmol H_2_O·m^−2^·s^−1^) recorded at three different phenological stages (booting, heading and milk maturity) in leaves of barley growing in control soil and *n*CeO_2_ and *n*TiO_2_-amended soil. Values are mean ± SE (*n* = 5). Same letters indicated no statistical difference between treatments at Tukey’s test. *** *p* < 0.001; ** *p* < 0.05; ns = not significant (*p* = 0.05).

Treatment	Booting	Heading					Milk Maturity		
	Amax	g_s_	Tr	Amax	g_s_	Tr	Amax	g_s_	Tr
*n*CeO_2_	0.0003 ***	0.0335	0.0047 **	ns	ns	ns	ns	ns	ns
*n*TiO_2_	0.0003 ***	0.0105 **	0.0105 **	ns	ns	ns	ns	ns	ns
*n*CeO_2_ × *n*TiO_2_	ns	ns	ns	ns	ns	ns	ns	ns	ns
Ctrl	20.4 ± 1.8 b	0.278 ± 0.05 ab	3.06 ± 0.64 b	19.4 ± 1.8 a	0.350 ± 0.06 a	3.24 ± 0.29 a	15.3 ± 1.6 a	0.298 ± 0.05 a	2.95 ± 0.30 a
Ce 500	25.7 ± 1.0 a	0.390 ± 00.5 a	5.36 ± 0.71 a	21.2 ± 1.3 a	0.254 ± 0.02 a	4.33 ± 0.58 a	14.4 ± 1.6 a	0.282 ± 0.04 a	2.53 ± 0.24 a
Ce 1000	19.4 ± 0.9 b	0.249 ± 0.03 b	3.23 ± 0.51 b	19.4 ± 1.7 a	0.220 ± 0.02 a	4.12 ± 0.33 a	17.8 ± 1.1 a	0.349 ± 0.05 a	3.15 ± 0.32 a
Ctrl	17.5 ± 1.8 b	0.205 ± 0.06 b	2.49 ± 0.26 b	21.6 ± 1.4 a	0.339 ± 0.04 a	3.79 ± 0.36 a	16.4 ± 1.3 a	0.229 ± 0.03 a	2.29 ± 0.21 a
Ti 500	22.8 ± 1.5 ab	0.287 ± 00.3 ab	3.73 ± 0.34 ab	18.5 ± 1.6 a	0.229 ± 0.03 a	4.04 ± 0.43 a	15.7 ± 1.4 a	0.327 ± 0.05 a	3.05 ± 0.29 a
Ti 1000	23.9 ± 1.0 a	0.387 ± 0.05 a	3.87 ± 0.45 a	18.6 ± 1.9 a	0.230 ± 0.03 a	3.64 ± 0.62 a	16.1 ± 1.8 a	0.357 ± 0.05 a	3.16 ± 0.30 a

**Table 2:** Ce concentration observed in roots, stems, leaves and kernels of barley grown in control soil and *n*CeO_2_ and *n*TiO_2_-amended soil. Values are mean ± SE. (*n* = 5). Same letters indicated no statistical difference between treatments at Tukey’s test (*p* ≤ 0.05).

Treatment	Ce Roots	Ce Stems	Ce Leaves	Ce Kernels
	(mg·kg^−1^)	(mg·kg^−1^)	(mg·kg^−1^)	(μg·kg^−1^)
Ctrl	3.30 ± 0.63 d	0.64 ± 0.12 c	0.73 ± 0.12 c	0.50 ± 0.19 a
Ce 500	45.3 ± 11.6 cd	1.38 ± 0.21 abc	1.62 ± 0.11 bc	0.87 ± 0.57 a
Ce 1000	96.9 ± 1.42 bc	1.75 ± 0.29 ab	3.03 ± 0.46 a	0.69 ± 0.36 a
Ti 500	14.0 ± 0.97 d	0.86 ± 0.29 bc	0.78 ± 0.11 c	0.98 ± 0.59 a
Ti 1000	19.1 ± 1.28 d	0.81 ± 0.21 bc	0.84 ± 0.09 c	1.22 ± 0.73 a
Ce 500/Ti 500	58 ± 9.61 cd	1.39 ± 0.24 abc	1.42 ± 0.15 bc	0.34 ± 0.12 a
Ce 500/Ti 1000	87 ± 5.60 bc	1.69 ± 0.23 ab	1.50 ± 0.08 bc	1.13 ± 0.52 a
Ce 1000/Ti 500	149 ± 19.4 ab	1.98 ± 0.19 a	1.76 ± 0.14 b	0.75 ± 0.41 a
Ce 1000/Ti 1000	164 ± 32 a	2.01 ± 0.17 a	1.79 ± 0.17 b	0.03 ± 0.01 a

**Table 3:** Ti concentration observed in roots, stems, leaves and kernels of barley grown in control soil and *n*CeO_2_ and *n*TiO_2_-amended soil. Values are mean ± S.E. (*n* = 5). Same letters indicated no statistical difference between treatments at Tukey’s test (*p* ≤ 0.05). d.l. = detection limit.

Treatment	Ti Roots	Ti Stems	Ti Leaves	Ti Kernels
	(mg·kg^−1^)	(mg·kg^−1^)	(mg·kg^−1^)	(μg·kg^−1^)
Ctrl	77 ± 3.19 a	0.26 ± 0.043 a	1.03 ± 0.06 a	1.39 ± 1.39 a
Ce 500	66.5 ± 5.15 a	0.19 ± 0.02 a	1.27 ± 0.32 a	0.48 ± 0.48 a
Ce 1000	63.9 ± 2.63 a	0.31 ± 0.02 a	1.31 ± 0.28 a	0.26 ± 0.18 a
Ti 500	66.7 ± 7.49 a	0.28 ± 0.03 a	1.39 ± 0.35 a	0.71 ± 0.61 a
Ti 1000	81.7 ± 4.96 a	0.39 ± 0.06 a	0.96 ± 0.09 a	<d.l.
Ce 500/Ti 500	63.9 ± 4.56 a	0.20 ± 0.09 a	1.33 ± 0.31 a	3.62 ± 2.60 a
Ce 500/Ti 1000	59.4 ± 7 a	0.22 ± 0.16 a	1.15 ± 0.27 a	8.14 ± 4.99 a
Ce 1000/Ti 500	69.1 ± 7.92 a	0.22 ± 0.07 a	1.07 ± 0.22 a	1.34 ± 1.3 a
Ce 1000/Ti 1000	68.4 ± 5.41 a	0.19 ± 0.04 a	0.89 ± 0.11 a	< d.l.
